# A unique foot-worn device for patients with degenerative meniscal tear

**DOI:** 10.1007/s00167-012-2026-2

**Published:** 2012-05-04

**Authors:** Avi Elbaz, Yiftah Beer, Ehud Rath, Guy Morag, Ganit Segal, Eytan M. Debbi, Daniel Wasser, Amit Mor, Ronen Debi

**Affiliations:** 1 AposTherapy Research Group, Herzliya, Israel; 2 Department of Orthopedic Surgery, Assaf HaRofeh Medical Center, Zerifin, Israel; 3 Department of Orthopedic Surgery, Sourasky Medical Center, Tel-Aviv, Israel; 4 Department of Orthopedic Surgery, Barzilay Medical Center, Hahistadrout St 2, 78278 Ashkelon, Israel

**Keywords:** Gait, Meniscal tear, Physical function, Pain, Osteoarthritis

## Abstract

**Purpose:**

The purpose of the current study was to assess the effects of a new foot-worn device on the gait, physical function and pain in patients suffering from knee osteoarthritis (OA) who had a low-impact injury to the medial meniscus causing a degenerative meniscal tear.

**Methods:**

A retrospective analysis of 34 patients with knee OA and a degenerative medial meniscal tear was performed. Patients underwent a gait evaluation, using an electronic walkway mat, and completed the SF-36 health survey and the WOMAC questionnaire at baseline and after 3 and 12 months of therapy. AposTherapy is a functional, biomechanical, non-invasive rehabilitation therapy consisting of a foot-worn device that is individually calibrated to each patient and is used during activities of daily living. Repeated-measures analyses were performed to compare gait parameters and self-evaluation questionnaires between baseline, and 3 and 12 months.

**Results:**

Significant improvements were found in gait velocity, step length and single-limb support of the involved knee following 12 weeks of therapy (all *p* < 0.01), alongside an improvement in limb symmetry. These results were maintained at the 12-month follow-up examination. Significant improvements were also found in all three domains of the WOMAC index (pain, stiffness and physical function) and in the SF-36 Physical Health Scale and the SF-36 Mental Health Scale (all *p* < 0.01).

**Conclusions:**

Patients with knee OA and a degenerative medial meniscal tear using a biomechanical foot-worn device for a year showed improvement in gait, physical function and pain. Based on the findings of this study, it can be postulated that this biomechanical device might have a positive effect on this population.

**Level of evidence:**

Therapeutic study, Level IV.

## Introduction

Meniscal tears are the leading cause of knee injury [34]. In the United States, 60 % of people over the age of 65 diagnosed with knee osteoarthritis (OA) suffer from chronic meniscal damage [13]. Meniscal tears have serious consequences as patients suffer from significant pain and a profound decline in their quality of life and physical function [34].

A variety of therapies exist to treat meniscal tears, ranging from pharmaceutical treatment [38] to physical therapy [15, 24] to surgery [2, 22, 30]. The most common invasive therapy has traditionally been meniscectomy [16], though the procedure has been reported to not halt the progression of cartilage destruction and premature OA [6, 29, 31], and it has even been suggested that the procedure may accelerate the development of OA [34–36]. Alongside this, Englund et al. [14] found that in knees without surgery, meniscal damage is a potent risk factor for the development of radiographic OA. In addition, recent work has found meniscectomy not to be superior to conservative treatment in regard to pain sensation, function and quality of life [24].

Gait analysis has been shown to be an objective measurement tool to assess pain, function and quality of life [9]. A common shortcoming of both surgical and non-surgical therapies (e.g. pharmaceutical management and physiotherapy) has been that proper limb symmetry and support during gait is rarely re-established [7, 37]. Earlier works have found that patients with abnormal gait patterns often suffer from impaired physical function [23] and pain [29]. Step length and single-limb support (SLS) are gait parameters that can demonstrate limb symmetry.

AposTherapy is a treatment that has been shown to improve gait patterns, physical function and pain in patients with orthopaedic pathologies, such as knee OA [3, 11, 20] and nonspecific low back pain [10]. These earlier works suggest that the changes in gait patterns and clinical findings seen with AposTherapy are due to small alterations in the centre of pressure that changes the vector trajectory and leads to reduced pain [18, 19, 21]. Based on AposTherapy principles, proper biomechanical alignment leading to reduced pain and neuromuscular training under controlled perturbation, it may be assumed that patients suffering from meniscal tears may benefit from this treatment and might avoid surgery.

The aim of the current study was to describe the effect of AposTherapy on gait patterns of patients with knee OA who had a low-impact injury to the meniscus causing a degenerative meniscal tear, alongside an analysis of the physical function, pain and quality of life condition throughout the therapy.

## Materials and methods

The study population composed of 34 patients (18 women). All patients were diagnosed with medial compartment knee OA by their physician and had a low-energy indirect injury to the knee, causing pain and functional limitation. Patients were diagnosed with a large complex medial meniscal tear related to the injury accompanied with bone bruise of the knee via magnetic resonance imaging (MRI) [5, 28]. Symptomatically, patients reported a sudden increase in their knee pain and limitation in function following the injury. The average age was 56.1 years (±11.1 years) with an average body mass index of 28.5 (±4.1 kg/m^2^). The patient’s functional severity was characterized using the functional classification scheme of Elbaz et al.: 6 % fell into Q1, 3 % fell into Q2, 26 % fell into Q3, 39 % fell into Q4 and 26 % fell into Q5. According to this classification, patients who fall into Q1 are characterized with poor walking abilities and high levels of pain and functional limitation, and patients who fall into Q5 are characterized with normal walking abilities and low levels of pain and functional limitation [12]. The patients sought medical care at AposTherapy Center in Herzliya, Israel. A retrospective search on the centre’s database for eligible patients was performed. Eligibility was defined according to the following criteria: diagnosed with knee OA according to American College of Rheumatology (ACR) clinical criteria [1], having a low-energy indirect injury to the medial meniscus of the knee diagnosed by MRI within the 3 months prior to arriving at the clinic, and completing a gait test and questionnaires at baseline and after 3 months and after 12 months. Exclusion criteria were (1) acute septic arthritis; (2) inflammatory arthritis; (3) corticosteroid injection within 3 months of the study; (4) avascular necrosis of the knee; (5) joint replacement; (6) neuropathic arthropathy; (7) history of pathological osteoporotic fracture; (8) symptomatic degenerative arthritis in lower limb joints other than the knees. The head researcher used these criteria to determine the inclusion or exclusion of patients from the existing database. The study was approved by the Helsinki committee of Assaf Harofeh Medical Center, Zerifin, Israel (ID no. 141/08). The study was registered in the NIH clinical trial registration system (No. NCT00767780).

### Gait analysis

The GaitMat™ system (E.Q., Inc. Chalfont, PA, USA) was used to measure gait spatiotemporal parameters. The computerized mat is an electronic walkway carpet that is 3.84 m long. The spatiotemporal characteristics are measured, processed and stored on a PC running the GaitMat software (version 2). The GaitMat™ system is a reliable tool to measure gait variables with significant accuracy, validly and reliably [4]. Temporal measurements taken simultaneously by the Gait Mat and Vicon had an ICC value of 0.99, indicating excellent reliability. Distance measurements taken by the two systems had an ICC value of 0.24, indicating poor to moderate reliability. The mean difference between distance measures was 11.7 mm, a difference that would be clinically significant only for support base measurements. The measurement accuracy is to the one decimal point [4].

### Physical function and pain assessment

The Western Ontario and McMaster Universities Osteoarthritis Index (WOMAC) was used to assess pain and physical function. It consists of a series of 24 self-administered questions measuring the pain, joint stiffness and physical function of a person with knee OA and is scored from 0 to 100 mm on a visual analogue scale (VAS), where 0 is the best and 100 is the worst total score. The validity and reliability of this questionnaire has been reported previously. The test–retest reliability Pearson’s correlation coefficients for the WOMAC items range from 0.55 to 0.78, all being significant [39]. The measurement accuracy is to the one decimal point.

The Short-Form Health Survey (SF-36) was used to measure patient quality of life. It consists of eight domains (pain, physical function, general health perceptions, role limitation due to physical problems, role limitation due to emotional problems, emotional well-being, social functioning and energy/fatigue), which can be tallied to create a Physical Health Scale and a Mental Health Scale. Each measurement is scored from 0 to 100, where 0 indicates the worst and 100 indicates the best patient health. The validity and reliability of this questionnaire has been reported previously. Reliability scores ranged from 0.76 to 0.93. The scores for all scales met the customary level of scale reliability [32]. The measurement accuracy is to the one decimal point.

## Apparatus

**Fig. 1:**
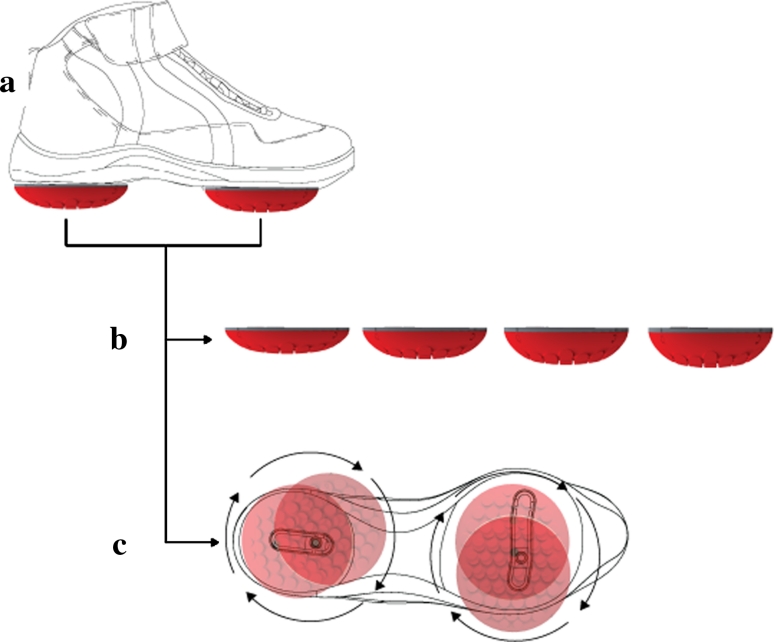
Apos biomechanical system. **a** Biomechanical device comprising two individually calibrated elements and a foot-worn platform. The elements are attached to under the hindfoot and forefoot regions of the platform. **b** The biomechanical elements are available in different degrees of convexity and resilience. **c** The specially designed sole of the platform includes two mounting rails and a positioning matrix to enable flexible positioning of each biomechanical element

The Apos system and AposTherapy were used in the study (APOS—Medical and Sports Technologies Ltd. Herzliya, Israel). The device is comprised of two bulbous-shaped biomechanical elements attached to the sole of a shoe (Fig. 1). One biomechanical element is located under the hindfoot, and the other is located under the forefoot region. The elements are attached to the subject’s foot via a platform in the form of a shoe. The platform is equipped with a specially designed sole, which consists of two mounting rails that enable flexible positioning of each element under each region. Each element can be individually calibrated to induce specific biomechanical challenges in multiple planes.

### Protocol

All patients underwent measurement of height and weight. Patients then underwent a computerized gait test. During the test, all patients walked barefoot at a self-selected speed. The following parameters were evaluated: velocity (cm/s), step length (cm) and SLS phase for each leg (% gait cycle, GC). In addition, patients completed the WOMAC and SF-36.

After the first gait test, the biomechanical device was individually calibrated to each patient by a physiotherapist specialized in AposTherapy methodology. Therapy was then initiated and continued on a daily basis for a period of 12 months. Patients were instructed to wear the device indoors as they perform their normal routines for an hour a day during the first week with an overall walking time of 10 min and to gradually increase walking time. All patients received a telephone call after the first and second weeks to verify compliance. A follow-up examination was conducted after 6 weeks in which patients were evaluated by the physiotherapist. After 3 and 12 months of therapy, patients underwent a gait test and completed the WOMAC and SF-36 questionnaires.

### Statistical analysis

Data were analysed with SPSS software version 19.0. The significance levels were set at 0.05. Data were presented as mean and standard deviation for continuous variables. Repeated-measures analyses were performed to compare gait parameters and self-evaluation questionnaires between baseline, and 3 and 12 months. Gait parameters and self-evaluation questionnaires following 1 year of therapy were presented by the mean and 95 % confidence intervals. Furthermore, repeated-measures analysis with one nested variable (gender) was conducted to demonstrate the differences in improvement between genders.

A change in gait measurements was considered to be clinically significant as long as it was accompanied by an improvement in the WOMAC index that qualified under the Outcome Measures in Rheumatology Clinical Trials (OMERACT)-OARSI guidelines for clinical improvement [28].

## Results

All patients complied with the study protocol, and none reported any adverse events that disqualified them from the study. One patient chose to undergo knee arthroscopy and was considered as a failure to treatment. Significant differences were found in the patients’ gait analysis after 12 months of therapy. Results are summarized in Table 1. In addition, significant improvement was noted in the levels of pain, function and stiffness, measured via the WOMAC index following 12 months of therapy (Table 2). The SF-36 Physical Health Scale and SF-36 Mental Health Scale (all *p* < 0.01) both improved significantly following 12 months of therapy (Table 2).

**Table 1:** Gait parameters changes following 12 months of therapy

	Baseline	3 months	12 months	p*
Velocity (cm/s)	97.4 (18.3) [90.9–103.9]	112.0 (18.3) [105.5–118.5]	111.8 (21.9) [104.0–119.5]	<0.001
Involved step length (cm)	55.2 (7.7) [52.5–58.0]	59.7 (7.6) [57.0–62.3]	59.1 (10.0) [55.5–62.6]	0.006
Uninvolved step length (cm)	55.8 (8.4) [52.9–58.8]	60.2 (7.9) [57.4–63.0]	60.3 (9.6) [56.9–63.7]	0.001
Involved single-limb support (% GC)	37.6 (2.3) [36.7–38.4]	38.8 (1.4) [38.3–39.3]	38.9 (1.7) [38.3–39.5]	0.001
Uninvolved single-limb support (% GC)	39.0 (2.2) [38.2–39.7]	39.2 (1.7) [38.6–39.9]	38.9 (2.1) [38.2–39.6]	n.s.

Results are presented as mean (SD) [95 % confidence interval]

A significant improvement was seen after 3 months of therapy as well as after 12 months of therapy compared to the baseline examination, except for SLS of the uninvolved limb

*GC* gait cycle

* *p* value was set to *p* < 0.05

**Table 2:** Changes in self-evaluation questionnaires following 12 months of therapy

	Baseline	3 months	12 months	p*
WOMAC index (0–100 mm)				
Pain	42.8 (21.5) [35.1–50.4]	22.7 (19.2) [15.9–29.5]	11.7 (14.0) [6.8–16.7]	<0.001
Stiffness	42.3 (26.0) [33.1–51.5]	20.5 (18.5) [13.9–27.0]	13.7 (16.6) [7.8–19.6]	<0.001
Function	36.9 (20.2) [29.7–44.0]	20.5 (17.4) [14.3–26.6]	13.2 (14.9) [7.9–18.5]	<0.001
SF-36 health survey (0–100)				
Physical function	49.2 (24.2) [40.6–57.8]	61.5 (21.4) [53.9–69.1]	67.8 (20.9) [60.5–75.3]	0.001
Pain	43.0 (20.8) [35.7–50.4]	61.1 (21.9) [53.3–68.8]	65.9 (23.8) [57.5–74.3]	<0.001
Limitation due to physical health	31.1 (38.5) [17.4–44.7]	58.3 (41.3) [43.7–73.0]	59.1 (39.4) [45.1–73.1]	0.001
Energy/fatigue	56.8 (18.4) [50.3–63.3]	64.8 (15.1) [59.5–70.2]	62.7 (16.4) [56.9–68.6]	0.04
Emotional well-being	71.3 (15.6) [65.8–76.8]	77.2 (12.8) [72.7–81.8]	75.9 (12.6) [71.4–80.4]	n.s.
Limitation due to emotional problems	55.6 (46.2) [39.2–71.9]	76.8 (35.8) [64.1–89.5]	70.7 (38.0) [57.2–84.2]	n.s.
Social functioning	68.2 (20.5) [60.9–75.5]	82.6 (16.8) [76.6–88.5]	86.0 (13.9) [81.1–90.9]	<0.001
General health	58.3 (17.9) [52.0–64.7]	66.0 (16.6) [60.1–71.9]	65.3 (12.4) [60.9–69.7]	0.03
Physical scale	47.7 (17.5) [41.5–53.9]	62.4 (17.9) [56.0–68.7]	64.2 (18.0) [57.8–70.6]	<0.001
Mental scale	62.0 (17.0) [56.0–68.1]	73.5 (13.5) [68.7–78.3]	72.1 (13.5) [67.3–76.9]	0.001

Results are presented as mean (SD) [95 % confidence interval]

* *p* value was set to *p* < 0.05. A significant improvement was seen after 3 months of therapy as well as after 12 months of therapy compared to the baseline examination, except for emotional well-being and limitation due to emotional problems

**Fig. 2:**
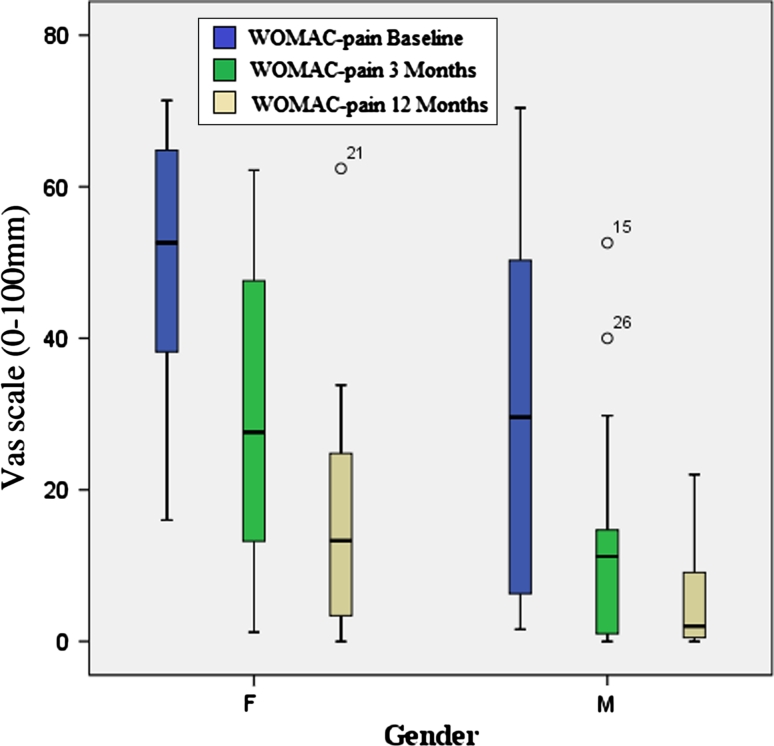
WOMAC-pain changes following 12 months of therapy in women and men. Women had significantly higher levels of pain compared to men at all time points. Both women and men reported significant reduction in pain following 12 months of therapy

**Fig. 3:**
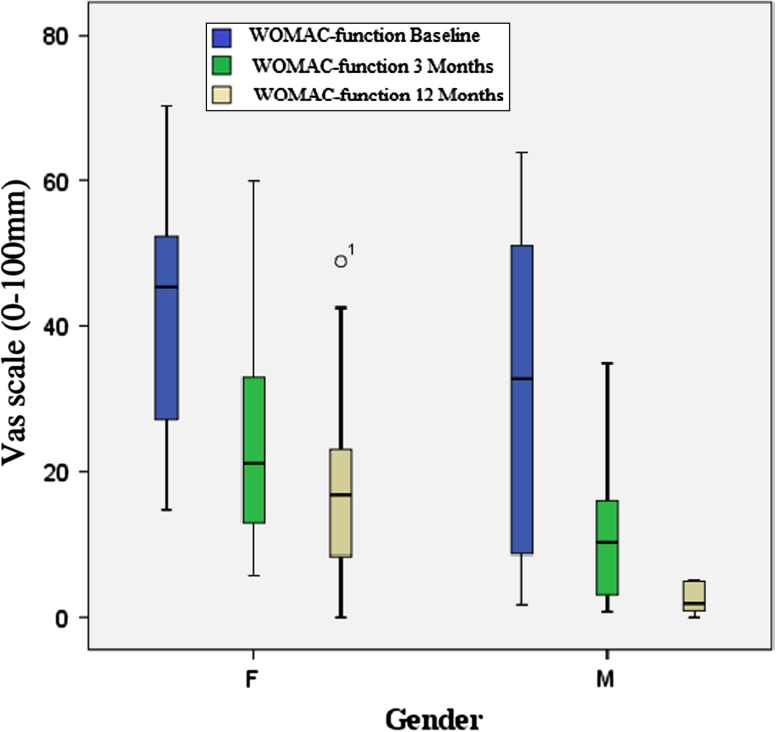
WOMAC-function changes following 12 months of therapy in women and men. Women had significantly higher levels of functional limitation compared to men at all time points. Both women and men reported significant improvement in function following 12 months of therapy

A comparison between men and women was made. Women showed significantly lower gait velocity (*p* = 0.011) and shorter step length (*p* = 0.001) compared to men at baseline and following 12 months of therapy. SLS did not differ between men and women. Both genders significantly increased their gait velocity, step length and SLS following 12 months of therapy. Results are summarized in Table 3. In regard to quality of life, no gender differences were noted in the physical health score or in the mental health score. Both genders reported an improvement in quality of life following 12 months of therapy (Table 3). Figures 2 and 3 illustrate gender differences in WOMAC-pain and WOMAC-function throughout the study period, respectively.

**Table 3:** Gender differences in gait parameters and self-evaluation questionnaire at baseline and following 3 and 12 months of therapy

	Males			Females		
	Baseline	3 months	12 months	Baseline	3 months	12 months
Velocity (cm/s)	105.4 (18.7) [94.7–115.4]	121.3 (16.4) [112.2–130.3]	119.6 (23.7) [106.5–132.7]	91.1 (15.7) [83.2–98.9]	104.3 (16.5) [96.1–112.5]	105.3 (18.5) [96.1–114.5]
Involved step length (cm)	59.5 (8.3) [54.9–64.1]	64.5 (7.1) [60.6–68.4]	63.4 (11.4) [57.1–69.7]	51.7 (5.0) [49.2–54.1]	55.7 (5.4) [53.0–58.1]	55.4 (7.2) [51.9–59.0]
Uninvolved step length (cm)	61.0 (8.5) [56.3–65.7]	65.6 (63.6) [61.9–69.2]	65.2 (11.0) [59.1–71.3]	51.6 (5.6) [48.8–54.4]	55.8 (5.9) [52.8–58.7]	56.2 (6.0) [53.2–59.2]
Involved single-limb support (% GC)	37.7 (2.6) [36.2–39.1]	39.0 (1.2) [38.4–39.7]	39.3 (1.1) [38.7–40.0]	37.5 (2.1) [36.4–38.5]	38.6 (1.6) [37.8–39.4]	38.5 (2.1) [51.9–59.0]
Uninvolved single-limb support (% GC)	39.6 (2.6) [38.2–41.1]	39.9 (1.2) [39.3–40.6]	39.3 (1.9) [38.3–40.4]	38.4 (1.6) [37.6–39.2]	38.7 (1.9) [37.7–39.7]	38.5 (2.2) [37.4–39.6]
SF-36 physical score	52.2 (21.2) [40.5–64.0]	65.9 (18.2) [55.8–75.9]	70.6 (16.1) [61.7–79.6]	43.9 (13.2) [37.3–50.5]	59.4 (17.6) [50.7–68.2]	58.8 (18.2) [49.7–67.9]
SF-36 mental score	67.1 (19.0) [56.1–77.6]	76.0 (14.2) [68.1–83.9]	76.9 (12.6) [69.9–83.8]	57.8 (14.3) [50.7–64.9]	71.4 (12.9) [65.0–77.6]	68.2 (13.3) [61.5–74.8]

Results are presented as mean (SD) [95 % confidence interval]

Significant differences between genders were found in velocity and step length at baseline and after 3 and 12 months of therapy. No significant gender differences were found in SLS and in SF-36 physical and mental scores. Both genders improved significantly in all measurements except for SLS of the uninvolved limb. A significant improvement was seen after 3 months of therapy and after 12 months of therapy compared to the baseline examination

*GC* gait cycle

*p* value was set to *p* < 0.05

A comparison between limbs was made for step length and SLS. No significant differences were found between involved step length and uninvolved step length at baseline and following therapy. Significant differences were seen between SLS of the involved limb compared to the SLS of the uninvolved limb at baseline (*p* = 0.013) and after 3 months of therapy (*p* = 0.023). There were no significant differences in SLS between limbs following 12 months of therapy.

## Discussion

The most important finding of this study was the improvement in gait patterns of patients with degenerative meniscal tears after 3 months of therapy with a foot-worn biomechanical device. This improvement was accompanied by improvements in symptoms and quality of life. The present findings have relevance for the treatment for meniscal tears in conjunction with knee OA and suggest a potent new therapy for patients suffering from degenerative meniscal tears.

The improvements in gait patterns have significant implications in regard to the ability of this therapy to re-establish proper limb symmetry. A common shortcoming of both surgical and non-surgical therapies has been that proper limb symmetry during gait is rarely re-established. Asymmetry in the lower extremity exposes the body to unusual and potentially excessive loads, which has a negative impact on walking and potentially increases the risk of the development of knee OA [6, 7, 26, 31, 37]. The fact that SLS of the non-injured knee did not change is an important finding as it was already in normal range. Based on this finding, limb asymmetry was minimized by increasing the weight-bearing time of the injured knee without changing the non-injured knee.

The functional improvement in gait dynamics was also supported by the self-evaluation questionnaire data, which found significant improvements in physical function and pain as defined via the WOMAC index and the SF-36, meeting the OMERACT OARSI criteria for clinical response to a treatment [33]. These findings are unique as they match, if not exceed, similar self-evaluative questionnaire results following other therapies [8, 25, 27]. Though earlier works have found improvement in the WOMAC index and the SF-36 of patients following surgical and pharmaceutical therapy, AposTherapy demonstrated a positive quantitative degree of improvement in physical function and pain following non-invasive therapy [8, 24, 27]. Lastly, though our findings have important clinical significance for the relatively short-term rehabilitation of meniscal tears, attention must be paid to the long-term implications. Numerous studies have related meniscus injuries with the development of knee OA [6, 26, 28]. The implemented therapy of this study has been shown to have a positive therapeutic effect on the gait pattern, physical function and pain of patients with knee OA [3, 11, 17, 20]. Therefore, it can be suggested that this treatment modality has the potential to be beneficial in two timeframes: the short-term rehabilitation of patients with meniscal tears and the long-term treatment for knee OA. These findings suggest that the use of this foot-worn biomechanical device may serve as an additional conservative treatment modality for patients with a degenerative meniscal tear and might reduce the need for surgery in these patients. Furthermore, since treatment is performed in the patient’s own environment while performing daily activities, high compliance is expected and the patient is driven to take responsibility for his recovery.

This study had some limitations. First, the present study lacked a control group; secondly, therapy did not commence immediately following the injury but within a 3-month time window. It is known, however, that in most cases, if a patient does not feel alleviation in pain within 3 months of injury, he or she will be recommended for surgery. It can be assumed that the patients in the present study received the standard care of treatment before commencing the biomechanical therapy and were recommended to undergo arthroscopy. Further studies should examine the effect of this therapy immediately after injury to see whether it can accelerate the rehabilitation period, as well as compare the therapy-compatible control group. Researchers should also consider comparing the outcomes of this conservative treatment with the outcomes of surgical procedures for meniscal injuries.

## Conclusions

Knee OA patients with degenerative meniscal tears treated with AposTherapy for 12 months demonstrated improved gait patterns (increased walking velocity, longer step length and higher SLS). Furthermore, patients also showed improved limb symmetry following the therapy. Finally, a statistically and clinically significant improvement was found in physical function and pain as measured by two different self-evaluation questionnaires, the WOMAC index and the SF-36.
